# Supplementary material for: Spray-Drying Encapsulation of the Live Biotherapeutic Candidate Akkermansia muciniphila DSM 22959 to Survive Aerobic Storage
Source: Pharmaceuticals (Basel). 2022 May 20;15(5):628. doi: 10.3390/ph15050628 (PMC9143277; doi:10.3390/ph15050628)
Supplement: Supplementary file 1 [file pharmaceuticals-15-00628-s001.zip › Supp_phamaceuticals.pdf]

Supplementary Information

Spray-drying encapsulation of the live biotherapeutic candidate *Akkermansia muciniphila* DSM 22959 to survive aerobic storage

Joana Cristina Barbosa[1], Diana Almeida[1], Daniela Machado[1]*, Sérgio Sousa[1], Ana Cristina Freitas[1†], José Carlos Andrade[2], Ana Maria Gomes[1]

[1] *Universidade Católica Portuguesa, CBQF - Centro de Biotecnologia e Química Fina – Laboratório Associado, Escola Superior de Biotecnologia, Rua Diogo Botelho 1327, 4169-005 Porto, Portugal;* [2] *TOXRUN – Toxicology Research Unit, University Institute of Health Sciences, CESPU, CRL, 4585-116 Gandra, Portugal*

*corresponding author: Daniela Machado, Universidade Católica Portuguesa, CBQF - Centro de Biotecnologia e Química Fina – Laboratório Associado, Escola Superior de Biotecnologia, Rua Diogo Botelho 1327, 4169-005 Porto, Portugal

E-mail address: dmachado@ucp.pt

Table S1. Viability of spray dried *Akkermansia muciniphila* DSM 22959 under aerobic storage conditions. The CFU counts correspond to three different matrices – SM: 10% skim milk; WPC: 10% whey protein concentrate (80 %); WPI: 10% whey protein isolate (90%); processed using 4 different combinations of inlet and outlet temperatures, as described. The resulting powders were aerobically stored, under controlled humidity, at 4 and 22 ºC, over a period of 28 days. Results are expressed in CFU/g ± standard deviation, unless stated otherwise; the CFU concentration initially inoculated is also indicated, as well as the concentration after the spray drying technique (corresponding to Day 0). BDL = Below Detection Limit.

Conditions	Initial (CFU/ml)		4 ºC				22 ºC			
		Day 0	7	14	21	28	7	14	21	28
SM (10 %)										
#1	$1.35 \pm 0.25 \times 10^{8}$	$3.23 \pm 0.34 \times 10^{7}$	$1.58 \pm 0.41 \times 10^{7}$	$1.90 \pm 0.40 \times 10^{7}$	$1.17 \pm 0.43 \times 10^{7}$	$1.87 \pm 0.34 \times 10^{7}$	$1.07 \pm 0.11 \times 10^{7}$	$2.90 \pm 0.65 \times 10^{6}$	$1.08 \pm 0.23 \times 10^{6}$	$7.83 \pm 1.86 \times 10^{5}$
#2		$1.20 \pm 0.22 \times 10^{8}$	$5.30 \pm 1.41 \times 10^{7}$	$5.58 \pm 1.60 \times 10^{7}$	$5.72 \pm 1.07 \times 10^{7}$	$4.82 \pm 1.16 \times 10^{7}$	$1.85 \pm 0.28 \times 10^{7}$	$1.33 \pm 0.49 \times 10^{7}$	$1.40 \pm 0.75 \times 10^{6}$	$1.17 \pm 0.25 \times 10^{6}$
#3		$1.10 \pm 0.20 \times 10^{8}$	$3.40 \pm 1.09 \times 10^{7}$	$4.15 \pm 0.47 \times 10^{7}$	$3.98 \pm 0.96 \times 10^{7}$	$4.53 \pm 0.85 \times 10^{7}$	$1.52 \pm 0.27 \times 10^{7}$	$4.05 \pm 1.57 \times 10^{6}$	$1.90 \pm 0.66 \times 10^{6}$	$1.12 \pm 0.46 \times 10^{6}$
#4		$3.83 \pm 0.33 \times 10^{7}$	$1.17 \pm 0.21 \times 10^{7}$	$1.08 \pm 0.28 \times 10^{7}$	$1.25 \pm 0.35 \times 10^{7}$	$1.08 \pm 0.34 \times 10^{7}$	$2.88 \pm 0.59 \times 10^{6}$	$1.45 \pm 0.46 \times 10^{6}$	$4.85 \pm 3.88 \times 10^{5}$	$4.25 \pm 0.82 \times 10^{5}$
WPC (10 %)										
#1	$1.16 \pm 0.11 \times 10^{9}$	$2.00 \pm 0.30 \times 10^{7}$	$7.67 \pm 0.75 \times 10^{6}$	$1.70 \pm 0.62 \times 10^{6}$	$3.32 \pm 1.52 \times 10^{6}$	$1.38 \pm 0.53 \times 10^{6}$	$6.33 \pm 1.25 \times 10^{5}$	BDL	BDL	BDL
#2		$2.40 \pm 1.02 \times 10^{7}$	$1.88 \pm 0.30 \times 10^{7}$	$1.22 \pm 0.66 \times 10^{7}$	$1.43 \pm 0.48 \times 10^{7}$	$9.33 \pm 2.52 \times 10^{6}$	$3.33 \pm 1.89 \times 10^{6}$	BDL	BDL	BDL

#3		3.83 ± 1.03 x 10^7	8.00 ± 3.58 x 10^6	BDL	BDL	BDL	5.33 ± 3.40 x 10^5	BDL	BDL	BDL
#4		9.70 ± 0.00 x 10^6	2.67 ± 0.94 x 10^6	BDL	BDL	BDL	7.00 ± 4.24 x 10^5	BDL	BDL	BDL
WPI (10 %)										
#1	5.57 ± 0.50 x 10^8	4.10 ± 0.71 x 10^6	2.55 ± 0.68 x 10^6	9.33 ± 3.40 x 10^5	4.80 ± 1.60 x 10^5	2.37 ± 0.85 x 10^5	2.67 ± 1.11 x 10^5	BDL	BDL	BDL
#2		1.47 ± 0.33 x 10^7	7.97 ± 4.89 x 10^6	2.72 ± 0.32 x 10^6	1.03 ± 0.64 x 10^6	2.25 ± 0.77 x 10^6	8.60 ± 1.74 x 10^5	BDL	BDL	BDL
#3		1.33 ± 0.47 x 10^7	1.03 ± 0.62 x 10^7	1.92 ± 0.56 x 10^6	1.83 ± 0.51 x 10^6	1.80 ± 0.34 x 10^6	9.80 ± 4.83 x 10^5	2.55 ± 0.87 x 10^5	1.83 ± 0.57 x 10^5	1.03 ± 0.39 x 10^5
#4		3.80 ± 0.59 x 10^6	1.92 ± 0.47 x 10^6	1.15 ± 0.27 x 10^6	4.52 ± 0.69 x 10^5	4.65 ± 0.73 x 10^5	2.04 ± 0.39 x 10^5	2.13 ± 0.65 x 10^4	3.44 ± 1.04 x 10^4	BDL

Table S2. Resistance to simulated GIT conditions of skim milk-incorporated, spray dried *Akkermansia muciniphila* DSM 22959 (SM). The samples were aerobically stored, at 4 ºC, for 60 days. The survivability to GIT conditions was evaluated every 2 weeks, following a standard protocol, as described [1], and CFU counts were evaluated every hour, with t = 0 corresponding to the *A. muciniphila* concentration at the beginning of the simulated GIT conditions; t = 2 to the end of the gastric phase; and t = 5, to the end of the intestinal phase.

Timepoints (h)	SM (CFU/g)				
	1	14	28	45	60
0	$3.52 \pm 0.00 \times 10^8$	$6.73 \pm 0.20 \times 10^7$	$4.69 \pm 0.97 \times 10^7$	$2.75 \pm 0.45 \times 10^7$	$2.60 \pm 0.24 \times 10^7$
1	$9.11 \pm 3.71 \times 10^7$	$1.62 \pm 0.21 \times 10^8$	$4.55 \pm 0.55 \times 10^7$	$2.07 \pm 1.28 \times 10^7$	$2.62 \pm 1.39 \times 10^7$
2	$1.30 \pm 0.23 \times 10^8$	$1.58 \pm 0.51 \times 10^8$	$3.63 \pm 0.77 \times 10^7$	$2.43 \pm 0.37 \times 10^7$	$2.42 \pm 0.69 \times 10^7$
3	$7.16 \pm 1.90 \times 10^7$	$4.17 \pm 0.43 \times 10^7$	$4.08 \pm 0.95 \times 10^7$	$3.27 \pm 1.04 \times 10^7$	$2.71 \pm 0.74 \times 10^7$
4	$5.50 \pm 0.74 \times 10^7$	$1.43 \pm 1.59 \times 10^7$	$4.61 \pm 1.75 \times 10^7$	$2.49 \pm 0.34 \times 10^7$	$3.06 \pm 1.61 \times 10^7$
5	$1.00 \pm 0.31 \times 10^8$	$4.54 \pm 0.16 \times 10^7$	$5.15 \pm 1.66 \times 10^7$	$2.28 \pm 1.15 \times 10^7$	$3.19 \pm 1.22 \times 10^7$

References

[1] Minekus, M.; Alminger, M.; Alvito, P.; Ballance, S.; Bohn, T.; Bourlieu, C.; Carrière, F.; Boutrou, R.; Corredig, M.; Dupont, D.; et al. A Standardised Static in Vitro Digestion Method Suitable for Food – an International Consensus. Food & Function 2014, 5, 1113–1124, doi:10.1039/C3FO60702J.
